# Biochemical assessment of patients following ketogenic diets for epilepsy: Current practice in the UK and Ireland

**DOI:** 10.1002/epi4.12371

**Published:** 2019-11-29

**Authors:** Natasha E. Schoeler, Zoe Simpson, Victoria J. Whiteley, Patty Nguyen, Rachel Meskell, Kathyrn Lightfoot, Kirsty J. Martin‐McGill, Simon Olpin, Fiona Ivison, the Ketogenic Dietitians Research Network (KDRN)

**Affiliations:** ^1^ UCL Great Ormond Street Institute of Child Health London UK; ^2^ Great Ormond Street Hospital for Children London UK; ^3^ Royal Manchester Children’s Hospital Manchester UK; ^4^ University of Salford Salford UK; ^5^ The National Centre for Neurology and Neurosurgery London UK; ^6^ Leeds Children’s Hospital Leeds UK; ^7^ University of Liverpool Liverpool UK; ^8^ University of Chester Chester UK; ^9^ Sheffield Children's Hospital Sheffield UK

**Keywords:** high fat, laboratory, low carbohydrate

## Abstract

**Objective:**

Biochemical assessment is recommended for patients prior to initiating and following a ketogenic diet (KD). There is no published literature regarding current practice in the UK and Ireland. We aimed to explore practice in comparison with international guidelines, determine approximate costs of biochemical testing in KD patients across the UK and Ireland, and promote greater consistency in KD services nationally.

**Methods:**

A survey was designed to determine the biochemical tests requested for patients at baseline, 3, 6, 12, 18, and 24 months + on KD. The survey was circulated to 39 centers across the UK and Ireland.

**Results:**

Sixteen centers completed the survey. Full blood count, electrolytes, calcium, liver function tests (LFTs), lipid profile, and vitamin D were requested at all centers at baseline, in keeping with international guidelines. Bicarbonate, total protein, and urinalysis were less consistently requested. Magnesium and zinc were requested by all centers, despite not being specifically recommended for pre‐diet evaluation in guidelines. Urea and electrolyte profiles and some LFTs were consistently requested at follow‐up, in accordance with guidelines. Other LFTs and renal tests, full blood count, lipid profile, acylcarnitine profile, selenium, vitamin D, and urinalysis were less consistently requested at follow‐up. The mean costs of the lowest and highest number of tests requested at baseline in our participating centers were £167.54 and £501.93; the mean costs of the lowest and highest number of tests requested at 3‐month follow‐up were £19.17 and £450.06.

**Significance:**

Biochemical monitoring of KD patients varies widely across the UK and Ireland and does not fully correspond to international best practice guidelines. With an ongoing drive for cost‐effectiveness within health care, further work is needed to streamline practice while ensuring patient safety.

**Key Points**

- Baseline tests are mostly in keeping with international guidelines, except for the addition of magnesium and zinc
- Not all tests were not consistently requested by all centers at follow‐up, despite recommendations
- Mean costs of baseline tests ranged from £167.54 to £501.93
- Mean costs of 3‐month follow‐up tests ranged from £19.17 to £450.06

## 1 INTRODUCTION

Ketogenic diets (KDs) are high‐fat, low‐carbohydrate, and moderate‐protein diets used as a treatment option for drug‐resistant epilepsy. KDs are the treatment of choice for neurometabolic disorders such as glucose transport type 1 deficiency syndrome^1^ and pyruvate dehydrogenase deficiency^2^ and are effective in reducing seizure frequency in approximately one third of patients with epilepsy.^3^

Ketogenic diets are inappropriate for some individuals, for example, with primary carnitine deficiencies and β‐oxidation defects, and thus, screening biochemical tests to rule out such disorders are a crucial part of pre‐diet assessment. KDs cause the body's metabolism to adjust, utilizing ketone bodies rather than glucose as its primary energy source. Due to stringent dietary restriction, individuals following a KD are often at risk of vitamin and mineral deficiencies,^4^, ^5^ and therefore, close biochemical monitoring is required to ensure nutritional adequacy and safety.

International consensus guidelines regarding optimal care of pediatric patients on KD therapies have been recently updated, including which biochemical tests (blood and urine) should be completed prior to diet initiation and during the treatment period.^6^ These follow on from KD care guidelines for resource‐limited countries published in 2015, including required and desired biochemical monitoring.^7^

Over the past two decades, the number of KD services in the UK and Ireland has increased from 22 to 39, with a concomitant surge in the number of patients on diet (from 101 in 2000^8^ to 754 in 2017).^9^ Centers in the UK and Ireland have local guidance for biochemical assessment and monitoring for KD patients but, to date, there has been no comparison nor consolidation of existing practices. Many of the biochemical tests required during KD treatment must be sent to specialist centers, further inflating costs and delays to treatment, conflicting with the current climate of the National Health Service (NHS), where services aim to be clinically and cost‐effective. The 2018 international recommendations involved a high proportion of non‐UK healthcare professionals from countries where costs are paid by insurance or by the patient privately, which could lead to disparities in practice.

We aimed to (a) explore current practice of biochemical testing in KD patients across the UK and Ireland in comparison with international guidelines, (b) determine approximate costs of biochemical testing in KD patients across the UK and Ireland, and (c) promote greater consistency in KD services nationally. To our knowledge, this is the first investigation of its kind. It is hoped that this work will help determine adherence to guidelines with regards biochemical monitoring of patients with epilepsy following a KD in the UK and Ireland, and whether action needs to be taken to streamline practice while ensuring patient safety and financial benefit.

## 2 METHODS

A survey was designed by the Ketogenic Dietitians Research Network (KDRN) (a consortium of KD Healthcare Professionals) to identify biochemical tests requested in patients commencing and following a KD for epilepsy and metabolic disorders in centers in the UK and Ireland. The ketogenic dietitians at each center were asked to list all biochemical investigations requested at baseline (pre‐diet), 3, 6, 12, 18, and 24 months post‐diet initiation during routine follow‐up (and other time points if applicable), as well as the frequency of biochemical follow‐up for patients on diet longer than two years (the point at which, routinely, patients and medical teams may consider discontinuing the diet). Centers were also invited to share the cost of each biochemical test requested as part of their KD service, if available, which provided an indication of the financial range anticipated for tests at both baseline and review.

The survey was disseminated via email to 39 services in the UK and Ireland. Following the initial email, two follow‐up emails were sent in an attempt to obtain more responses. All answers were pseudo‐anonymized, and results were compared to the laboratory assessments recommended in international best practice guidelines,^6^ as outlined in Table 1.

**Table 1:** Laboratory assessments recommended as part of pre‐diet evaluation and follow‐up visits in international best practice guidelines^6^

Laboratory assessment prior to ketogenic diet initiation	Laboratory assessment during ketogenic diet treatment
Complete blood count with platelets	Complete blood count with platelets
Electrolytes (including bicarbonate, total protein, calcium)	Electrolytes (including bicarbonate, total protein, calcium)
Liver and kidney tests (including albumin, blood urea nitrogen, creatinine)	Liver and kidney profile (including albumin, blood urea nitrogen, creatinine)
Fasting lipid profile	Fasting lipid profile
Serum acylcarnitine profile	Free and total carnitine
Vitamin D level	Vitamin D level
Urinalysis	Urinalysis
Antiseizure drug levels (if applicable)	Antiseizure drug levels (if applicable)
Urine organic acids (if diagnosis unclear)	Selenium level
Amino acids (if diagnosis unclear)	Optional
	Beta‐hydroxybutyrate (BOH) level
	Urine calcium and creatinine
	Zinc, copper levels

## 3 RESULTS

Sixteen centers completed the survey: 15 pediatric centers (of which 14 were NHS) and one non‐NHS joint adult and pediatric center.

The number of patients referred annually for KD treatment in each of these centers, the patient population (pediatrics or adults), and the type of center (primary, secondary, or tertiary care) are outlined in Table 2.

**Table 2:** Key characteristics of participating centers

Center ID	Number of referrals (2016‐17)^a^	Patient population	Level of care
Center 1	9	Pediatrics	Tertiary
Center 2	52	Pediatrics	Tertiary
Center 3	17	Pediatrics	Tertiary
Center 4	25	Adults	Tertiary
Center 5	53	Pediatrics	Tertiary
Center 6	76	Pediatrics	Tertiary
Center 7	42	Pediatrics and adults	Tertiary
Center 8	34	Pediatrics	Tertiary
Center 9	45	Pediatrics	Tertiary
Center 10	30	Pediatrics	Tertiary
Center 11	17	Pediatrics	Tertiary
Center 12	24	Pediatrics	Tertiary
Center 13	23	Pediatrics	Tertiary
Center 14	45	Pediatrics	Tertiary
Center 15	18	Pediatrics	Tertiary
Center 16	39	Pediatrics	Tertiary
Center 17	12	Pediatrics	Tertiary
Center 18	21	Adults	Tertiary
Center 19	30	Pediatrics	Tertiary
Center 20	7	Pediatrics	Tertiary
Center 21	20	Pediatrics	Tertiary
Center 22	6	Pediatrics	Secondary

a From July 1, 2016, to June 30, 2017 (Whiteley et al^10^).

### 3.1 Current practice and comparison to international guidelines

A total of 63 different biochemical tests were requested across the participating centers. Table 3 outlines recommended tests, clustered into clinical groups, and lists which groups of tests were requested by all, by 90%‐99%, by 75%‐90%, by 50%‐75%, and then by <50% of participating centers. A list of all tests (ungrouped) and the percentage of centers that requested each test at each time point can be found in the Supplementary Table S1.

**Table 3:** Proportion of participating centers requesting biochemical tests recommended for ketogenic diet patients^6^

	Full/complete blood count	Electrolytes	Total protein	Calcium	Liver function tests	Renal profile	Lipids	Acylcarnitine Profile^a^	Vitamin D	Urinalysis	Selenium
Baseline	All	All	50%‐74%	All	All	75%‐90%^c^	All	All	All	<50%	n/a
3‐month follow‐up^b^	All	All	50%‐74%	75%‐90%	75%‐90%^d^	75%‐90%^c^	All	75‐90%	75%‐90%	<50%	50%‐75%
6‐month follow‐up	All	All	50%‐74%	90%‐99%	90%‐99%^d^	75%‐90%^c^	All	75%‐90%	75%‐90%	<50%	75%‐90%
12‐month follow‐up	All	All	50%‐74%	All	All	75%‐90%^c^	All	75%‐90%	90%‐99%	<50%	90%‐99%
18‐month follow‐up	90%‐99%	All	50%‐74%	90%‐99%	75%‐90%^d^	75%‐90%^c^	90‐99%	50%‐74%	75%‐90%	<50%	75%‐90%
24‐month follow‐up	All	All	50%‐74%	All	All	75%‐90%^c^	All	75%‐90%	All	<50%	90%‐99%

Note

Antiseizure drug levels are not included, despite recommendations in guidelines, as these are only applicable to certain patients.

Electrolytes (sodium, potassium); liver function tests (albumin, alanine aminotransferase (ALT), alkaline phosphatase (ALP), bililrubin); renal profile (urea [referred to as blood urea nitrogen in Kossoff et al. 2018], creatinine, bicarbonate); lipids (total cholesterol and triglycerides as minimum); vitamin D (total 25hydroxy vitamin D).

a In international guidelines, acylcarnitine profile is recommended at baseline, and free and total carnitine at review. These have been grouped together under acylcarnitine profile, as this is the standard investigation in the UK/Ireland, which includes reporting of free carnitine and the full range of acylcarnitine species.

b Two centers did not routinely request any tests at 3‐month follow‐up, 1 center did not request tests at 18‐month follow‐up, and 1 center requested tests at 3 months and then 6‐monthly thereafter; these centers have been excluded at these time points.

c All centers requested urea and creatinine at baseline, and at each follow‐up; 75‐90% requested bicarbonate at baseline and at each follow‐up.

d ALT and ALP requested by all centers at every follow‐up; albumin requested by all centers at every follow‐up except for 18 months.

### 3.2 Baseline monitoring

Full blood count, electrolytes, calcium, liver function tests (LFTs), lipid profile, and vitamin D were requested at all centers at baseline, in keeping with international guidelines (Table 3). Bicarbonate, total protein, and urinalysis are recommended in international guidelines but were not consistently requested by our participating centers. Magnesium and zinc were also requested by all centers, despite not being specifically mentioned for pre‐diet evaluation in international guidelines.

### 3.3 Follow‐up monitoring

Twelve centers requested biochemical tests routinely at 3, 6, 12, 18, and 24 months post‐diet initiation; two centers did not request any tests at 3 months, one center that did not request any tests at 18 months, and one center requested tests at 3 months and then 6‐monthly thereafter (testing requested at 3,9, and 15 months post‐diet initiation).

Urea, creatinine and electrolytes, alanine aminotransferase (ALT), and alkaline phosphatase (ALP) were requested by all centers at 3‐, 6‐, 12‐, 18‐, and 24‐month follow‐up (centers that routinely requested biochemical tests at these respective time points). Full blood count, lipid profile, and albumin were requested by each of our centers at every review except for the 18‐month point. Other components of renal profile and liver function tests, acylcarnitine profile, selenium, vitamin D, and urinalysis were less consistently requested at follow‐up (Table 3).

13/16 (81%) centers requested non‐fasting lipid profiles, both at baseline and review, despite recommendations for a fasting lipid profile. Seven of these 13 requested non‐fasting lipid profiles, but would repeat in a fasted state if initial results were abnormal.

### 3.4 Long‐term follow‐up

For those centers following patients up for more than 2 years: 10/16 (63%) centers requested 6‐monthly monitoring for patients following a KD, in keeping with guidelines advising 6‐monthly visits after following a KD for 1 year. 5/16 (33%) centers requested yearly monitoring and 1/16 (6%) had no protocol.

### 3.5 Cost implications

The mean costs of the lowest and highest number of tests requested at baseline in our participating centers were £167.54 and £501.93; the mean costs of the lowest and highest number of tests requested at 3‐month follow‐up were £19.17 and £450.06. For comparison, the mean cost per visit of all biochemical tests recommended in international guidelines was £108.96 at baseline and £126.09 at review. The minimum, maximum, and mean cost per test can be found in Table 4.

**Table 4:** Minimum, maximum, and mean costs of biochemical tests in participating centers

Analyte(s)	Minimum (£)	Maximum (£)	Mean (£)
Urea and Electrolytes (U/E)^a^	1.45	12	5.4
Bone profile^a^	0.99	12	4.88
Liver function Tests^a^	0.66	12	4.67
Lipid Profile	0.52	12	5.23
Beta‐hydroxybutyrate	6.24	77.95	27.85
Magnesium^a^	0.24	14.32	5.22
Zinc^b^	7.13	21.4	13.06
Selenium^b^	10	26.54	17.13
Copper^b^	7.13	14.32	11.15
Glucose	0.24	5.87	2.66
Acylcarnitine Profile^c^	32.24	100	69.8
Amino acid profile^c^	82	100	103.14
Urine calcium creatinine ratio	2.66	14.32	7.07
Full blood count (for Hb)	1.34	7.99	3.87
B12	2.35	14.32	7.54
Folate	2.49	12	6.26
Ferritin	2.09	12	5.77
Vitamin A^d^	11.5	20.98	18.18
Vitamin E^d^	11.5	20.98	18.18
25‐hydroxy vitamin D3^e^	4.42	26.05	14.79
Clotting Screen	2.74	14.32	6.745
Urine Ketones (urine dipsticks)	0.11	0.26	0.315
Non‐esterified fatty acids	77.95	77.95	£77.95
Acetoacetate	6.24	6.24	6.24
Thyroid function test	5.29	12	8.645
Amylase	4.63	4.63	£4.63
Urine organic acids	45.5	58.24	51.87
TOTAL	£329.65	£710.68	£508.245

a These components can be combined into a full profile, which may be lower cost than individual sets.

b Trace elements may be able to be analyzed on a single sample in some centers, with a lower cost than the individual metals.

c These tests are only carried out at highly specialized laboratories and thus cost more due to limited availability and high degree of technical skill in carrying out the assay and interpreting the results.

d Vitamin A and E are usually analyzed together and should be at lower cost than when requested individually.

e The varied technology available to measure 25‐hydroxy vitamin D3 greatly affects the cost of the test.

## 4 DISCUSSION

Our study illustrates that biochemical assessment and monitoring of KD patients with epilepsy vary widely across the UK and Ireland and do not fully correspond to international best practice guidelines.^6^ This variability is reflected in the associated costs of biochemistry testing. To our knowledge, there are no other previously published works outlining which biochemical tests are requested in KD patients in the UK and Ireland and their financial impact.

Variability of practice is inevitable, due to differing patient populations in each center and the acute needs of individuals, particularly in the complex cohort that commence dietary therapy for refractory epilepsy. However, the level of variability among our participating centers seems striking. This may be partially explained by the fact that the expansion of KD services in the UK and Ireland is a recent and somewhat sporadic phenomenon.^9^, ^10^ Only in recent years have technological advances and the creation of national groups, such as KDRN, facilitated liaison across participatory centers, promoting communication, and sharing of resources. Research study protocols, such as those from the original randomized controlled trial at Great Ormond Street^11^ and Ketogenic Diet in Infants with Epilepsy (KIWE),^12^ may also influence what tests are requested at participating centers.

Our costing results, although approximate, indicate that biochemical testing for KD patients can have a substantial financial impact on services, as well as highlighting the variability between centers. The final cost to an individual center will vary, as large teaching hospitals can often benefit from lower costings due to higher workload and are more likely to have specialist tests available on site. Between hospital laboratories, the items included in a profile vary. In addition, the type of technology used (eg high‐throughput minimal intervention automated analyzers versus mass spectrometry for 25‐hydroxy vitamin D3) and, in some cases, the interpretation of the laboratory price list can also impact the final cost: Some centers may ask for the cost to measure a set of electrolytes, liver function tests, and a bone profile, whereas asking for a “full profile” should cost slightly less due to the overlap in tests. Notwithstanding these caveats, a difference of £334.39 between minimum and maximum requested baseline tests and £430.89 for 3‐month review tests in our participating centers is noteworthy.

Any “lesser” costs in KD laboratory monitoring need to be balanced against the possible increased risk of complications, with associated costs. Even the cost of “complete” KD monitoring may be less than treatment with a new antiepileptic drug, which can cost up to approximately £100/month,^13^ as well as the costs implicated in seizure‐related complications. On the other hand, it may not be appropriate to test for each recommended parameter at every review, such as vitamin D, due to the time taken for changes to take effect.^14^, ^15^

Magnesium and zinc were requested by all our participating centers at baseline, despite not being included in international best practice guidelines for pre‐diet evaluation. This may represent a cost saving *if* unnecessary in most patients. No report of zinc deficiencies in individuals following a KD has been identified, although classical KDs with a 2:1 ratio or higher fail to meet the dietary reference intake for zinc, despite “selection of nutrient dense foods”.^16^ One may argue that if mentioned as “optional” to measure at review, as in international guidelines, baseline assessment of zinc would also be appropriate. Mean plasma magnesium levels have been found to decrease in children on the classical KD,^4^ and the diet has been shown to provide suboptimal magnesium levels.^16^ Intakes of zinc and magnesium may be suboptimal even prior to KD initiation: 3%‐27% and 0%‐50% of the UK population surveyed in the latest National Diet and Nutrition Survey (including males and females across all age groups above 1.5 years) do not meet the lower reference nutrient intake (RNI) for zinc and magnesium, respectively.^17^

The discrepancies between which tests were requested at all review appointments in our centers compared to international recommendations may be predominantly cost‐driven, particularly considering that the 2018 guidelines involved a high proportion of non‐UK healthcare professionals from countries where costs are paid by insurance or by the patient privately, compared to the government‐funded UK National Health Service, which could potentially be considered “resource‐limited.” Furthermore, while NICE recommends KDs for pediatric refractory epilepsy,^18^ it does not suggest recommendations for monitoring and so there is no UK cost‐effective reference guidelines. In previous guidelines issued for resource‐limited regions, bicarbonate was deemed mandatory at baseline and review, and urinalysis and lipid profile were mandatory at review.^7^

Published reports of abnormalities in individuals following KDs may provide guidance as to whether it is necessary to request the “missing” parameters at each review in UK and Ireland centers. Besides dyslipidemia, which is one of the most well‐cited (although often transient) biochemical side effects of KDs, occurring in approximately 12% of children studied prospectively on a KD,^19^ reports of abnormalities of other parameters are uncommon. Individuals following a KD have been shown to have reduced serum 25‐hydroxyvitamin D concentration (as were individuals solely on antiepileptic drugs) and reduced bone mass (to a greater extent than in individuals solely on drug therapy)^20^; another study found 25‐hydroxyvitamin D levels (which were mostly low at diet initiation) to improve initially on commencement of a KD, including supplementation, but to decline after three months.^21^ Selenium deficiency has been reported in 66 individuals on KD treatment,^22^, ^23^, ^24^, ^25^ associated with cardiomyopathy in two of these patients^23^, ^25^ and sudden cardiac death in another two.^24^ A trend of decreasing plasma selenium was noted in participants of the original randomized controlled trial at GOSH, with a significant decrease between baseline and six months in children on the classical KD, although mean plasma selenium was maintained within the GOSH reference ranges.^4^

In view of the possible association of selenium with cardiac abnormalities, monitoring of selenium seems significant, although prolonged QT interval has also been reported in three cases following a KD in the absence of selenium deficiency.^26^ The frequency of testing at review, particularly in the 12‐ to 24‐month follow‐up period, may need revisiting specifically for the UK and Ireland in order to balance clinical safety with the potential financial/logistical constraints of 6‐monthly testing.

This study has several limitations. Only 16 of the 39 centers that (to our knowledge) practice KDs within the UK and Ireland volunteered to answer the survey; practice in the other centers remains unknown. The survey was not validated and could be subject to reporter error. Our cost estimations would also improve in accuracy with greater center participation. A follow‐up study to assess whether rate of complications is correlated with frequency and “completeness” of laboratory monitoring would be pertinent.

Biochemical monitoring of KD patients varies widely across the UK and Ireland and does not fully correspond to international best practice guidelines. With an ongoing drive for cost‐effectiveness within the NHS, further work is needed to streamline practice while ensuring patient safety, both for financial benefit without clinical compromise for patients, perhaps with the creation of nationwide‐specific guidelines. Further research into biochemical monitoring of KDs worldwide would be of interest to compare to practice in the UK and Ireland.

## CONFLICTS OF INTERESTS

Matthew's Friends Charity, Nutricia Advanced Medical Nutrition and Vitaflo (International) Ltd sponsored meetings for KDRN, one of which was used to formulate this project. NES is supported by a research grant from Vitaflo (International) Ltd. KJM‐M received a PhD studentship from Vitaflo (International) Ltd. The remaining authors have no conflicts of interest. No funding is declared. We confirm that we have read the Journal's position on issues involved in ethical publication and affirm that this report is consistent with those guidelines.
